# Draft Genome Sequences of Two Commensal *Enterococcus cecorum* Strains Isolated from Chickens in Belgium

**DOI:** 10.1128/genomeA.01108-15

**Published:** 2015-09-24

**Authors:** Beata Dolka, Filip Boyen, Patrick Butaye, Rikke Heidemann Olsen, Ida Cecilie Naundrup Thøfner, Jens Peter Christensen

**Affiliations:** a Department of Pathology and Veterinary Diagnostics, Faculty of Veterinary Medicine, Warsaw University of Life Sciences, SGGW, Warsaw, Poland; b Department of Pathology, Bacteriology and Avian Diseases, Faculty of Veterinary Medicine, Ghent University, Merelbeke, Belgium; c Department of Biosciences, Ross University, School of Veterinary Medicine, Basseterre, St. Kitts and Nevis, West Indies; d Department of Veterinary Disease Biology, Faculty of Health and Medical Sciences, University of Copenhagen, Frederiksberg, Denmark

## Abstract

Here, we report the draft genome sequences of two commensal *Enterococcus cecorum* strains (1710s23 and 1711s24), cultivated from the ceca of healthy laying hens originating from different farms in Belgium.

## GENOME ANNOUNCEMENT

*Enterococcus cecorum* is a normal inhabitant of the gastrointestinal flora of mammals and birds (1). It is the most frequently occurring enterococcal species in the intestine of adult chickens (2). Recently, several *E. cecorum*–related clinical cases in poultry have been reported worldwide (3–6).

Here, we report two draft genome sequences of commensal *E. cecorum* strains that were isolated from the ceca of 2 laying hens from different farms in Belgium. The chickens were submitted in August 2009 to the Department of Pathology, Bacteriology, and Avian Diseases, University of Ghent. Birds did not show clinical signs or necropsy findings associated with disease. *E. cecorum* was not isolated from any of the extraintestinal organs. For each bird, cecal content was collected aseptically and plated on sheep blood agar supplemented with colistin and nalidixic acid (Biomedia Ltd., Canada) and incubated for 24 to 48 h at 35°C in 5% CO_2_. Isolates were recovered from one bird per farm. The identification was performed by API 20S (bioMérieux, France) and further confirmed by sequencing of the 16S rRNA genes (7) and alignment with the sequences of the reference strains. The recovered *E. cecorum* isolates were assigned as control (intestinal) isolates and used for comparison studies (8). PFGE (Pulsed Field Gel Electrophoresis) revealed a large genetic diversity of *E. cecorum* from noninfected control chickens (8).

Genomic DNA of *E. cecorum* strains 1710s23 and 1711s24 was extracted using the DNeasy blood and tissue kit (Qiagen, USA). DNA quantity and quality were assessed using Nanodrop spectroscopy (Thermo Scientific, USA). Genomes were sequenced by the Illumina paired-end method (MiSeq 150) using a paired-end library with an average read length of 2 × 150 bp. Reads were *de novo* assembled using the CLC Genomics Workbench version 7.0. The draft genome assembly of 1710s23 is 2.26 Mb, comprising 28 contigs (range, 271 to 424,975 bp), with an average length (*N*_50_) of 170,910 bp and 36.4% G+C content. Similarly, the 1711s24 genome has 28 contigs (range, 1,407 to 424,966 bp), with a total genome size of 2.30 Mb, an *N*_50_ of 170,921 bp, and 36.2% G+C content. Both genomes were submitted to Rapid Annotations using Subsystems Technology (RAST) (9) and the NCBI Prokaryotic Genome Automatic Annotation Pipeline (PGAAP, http://www.ncbi.nlm.nih.gov/genome/annotation_prok) (10) for annotation. The 1710s23 genome is estimated to contain 2,247 genes (12 rRNAs, 48 tRNAs) and 2,099 expected protein-coding sequences (CDSs). The 1711s24 genome contains 2,295 genomic features consisting of 2,143 CDSs (predicted), 46 tRNAs, and 10 rRNAs. No virulence factors were found by using the VirulenceFinder version 1.4 server (11).

To the best of our knowledge, this is the first genome report of commensal *E. cecorum* isolated from commercial laying hens in Europe. The data reported here may be useful in the investigation of genome variability of *E. cecorum* and for future comparative genomic studies on the pathogenicity of *E. cecorum*.

### Nucleotide sequence accession numbers.

The whole-genome shotgun projects have been deposited at DDBJ/EMBL/GenBank under the accession numbers LDOW00000000 (1710s23) and LDOX00000000 (1711s24). The versions described in this paper are the first versions, LDOW01000000 and LDOX01000000, respectively.
